# Stability of glucagon-like peptide 1 and glucagon in human plasma

**DOI:** 10.1530/EC-14-0126

**Published:** 2015-02-04

**Authors:** Nicolai J Wewer Albrechtsen, Monika J Bak, Bolette Hartmann, Louise Wulff Christensen, Rune E Kuhre, Carolyn F Deacon, Jens J Holst

**Affiliations:** 1 NNF Center for Basic Metabolic Research and Department of Biomedical Sciences, Faculty of Health Science, University of Copenhagen, Blegdamsvej 3B, 12.2, DK-2200 Copenhagen N, Denmark; 2 Department of Science, Faculty of Health Science, University of Copenhagen, Blegdamsvej 3B, 12.2, DK-2200 Copenhagen N, Denmark

**Keywords:** glucagon, glucagon-like peptide-1, degradation, storage, samples handling

## Abstract

To investigate the stability of glucagon-like peptide 1 (GLP-1) and glucagon in plasma under short- and long-term storage conditions. Pooled human plasma (*n*=20), to which a dipeptidyl peptidase 4 (DPP4) inhibitor and aprotinin were added, was spiked with synthetic GLP-1 (intact, 7–36NH_2_ as well as the primary metabolite, GLP-1 9–36NH_2_) or glucagon. Peptide recoveries were measured in samples kept for 1 and 3 h at room temperature or on ice, treated with various enzyme inhibitors, after up to three thawing–refreezing cycles, and after storage at −20 and −80 °C for up to 1 year. Recoveries were unaffected by freezing cycles or if plasma was stored on ice for up to 3 h, but were impaired when samples stood at RT for more than 1 h. Recovery of intact GLP-1 increased by addition of a DPP4 inhibitor (no ice), but was not further improved by neutral endopeptidase 24.11 inhibitor or an inhibitor cocktail. GLP-1, but not glucagon, was stable for at least 1 year. Surprisingly, the recovery of glucagon was reduced by almost 50% by freezing compared with immediate analysis, regardless of storage time. Plasma handling procedures can significantly influence results of subsequent hormone analysis. Our data support addition of DPP4 inhibitor for GLP-1 measurement as well as cooling on ice of both GLP-1 and glucagon. Freeze–thaw cycles did not significantly affect stability of GLP-1 or glucagon. Long-term storage may affect glucagon levels regardless of storage temperature and results should be interpreted with caution.

## Introduction

Glucagon-like peptide 1 (GLP-1) and glucagon arise from differential processing of the glucagon precursor, proglucagon (PG) (1). Both peptides are important for normal glucose homeostasis, which focused interest on their potential role in the pathophysiology of type 2 diabetes mellitus (T2DM), and has led to the development of GLP-1-based therapies for treatment of T2DM (2). These comprise injectable GLP-1 receptor agonists as well as orally available inhibitors of dipeptidyl peptidase 4 (DPP4), the enzyme responsible for the initial rapid degradation of the intact (7–36NH_2_) GLP-1 to its metabolite (9–36NH_2_) (2, 3). The closely related peptide, glucagon, is not a substrate for DPP4 under physiological circumstances (4).

Measurement of GLP-1 and glucagon levels is increasingly being included as endpoints in study design (∼500 diabetes-related clinical trials having GLP-1 or glucagon as outcome measures – see clinicaltrials.gov). The stability of these peptides in plasma may be affected by sampling and storage conditions, but whether these influence measured concentrations has not yet been investigated. Our study therefore seeks to define optimal conditions for handling of human plasma samples with regards to both short-term handling procedures and long-term storage, as well as the influence of repeated thawing–refreezing cycles. We used synthetic peptides to evaluate their stability, but it may be questioned whether their stability reflects the stability of the endogenous, immunoreative peptides. Both glucagon and GLP-1 exhibit molecular heterogeneity, both due to differential processing of their common precursor and post-secretory metabolism. Our data therefore mainly apply to the structures normally regarded as ‘glucagon’ (proglucagon 33–69) and ‘GLP-1’ (proglucagon 78–106 amide). However, in this study use of assays covering both the N- and the C-terminus of the molecules (5, 6) ensures that our results are also applicable to the additional circulating molecular entities.

## Materials and methods

### Sample preparation

Blood from 20 healthy volunteers (procedure approved by the local ethical committee and conducted according to the principles of The Helsinki Declaration) was collected into pre-chilled EDTA tubes and centrifuged for 20 min at 2261 ***g*** at 4 °C. Plasma was separated immediately and pooled. Next, aprotinin (Trasylol 10000 KIE/ml; Bayer Health Care AG 51368; final concentration 500 KIE/ml) and a DPP4 inhibitor (valine pyrrolidide, a gift from Novo Nordisk A/S, Bagsværd, Denmark; final concentration 0.01 mmol/l) were added to the plasma pool, which was kept at low temperature (ice bath or cold room) at all times. Stock solutions of synthetic glucagon 1–29 (Bachem, Bubendorf, Switzerland, Cat. No. H-6790), GLP-1 7–36NH_2_ (Bachem; Cat. No. H-6795.0500) and GLP-1 9–36NH_2_ (Bachem; Cat. No. H-4012.0001) were prepared, and their concentrations were verified by quantitative amino acid analysis (QAAA; duplicate determination) and RIA using mid-region, processing-independent antisera (codename 4304 for glucagon and 2135 for GLP-1) as previously described (7, 8). The results were as follows: glucagon 1–29 (RIA 95% of expected concentration; QAAA 92%), GLP-1 7–36NH_2_ (RIA 51% of expected concentration; QAAA 49%), and GLP-1 9–36NH_2_ (RIA 77.2% of expected concentration; QAAA 71%). Known amounts of each peptide (corrected according to these results) were added to plasma aliquots to increase concentrations by 0 (solvent only), 10, and 40 pmol/l, respectively, to give a total of nine portions of spiked plasma, whereafter each portion was aliquoted (*N*=6–8) into separate tubes and processed to different short-term or long-term storage conditions, as described below.

### The effect of short-term storage conditions on the stability of GLP-1 and glucagon

The aliquots (*N*=6–8) of spiked plasma were then: i) immediately extracted with ethanol (final concentration 70%, for this treatment we used *N*=21); ii) subjected to one freeze–thaw cycle (placed at −20 °C for ∼1 h followed by thawing in an ice bath) and extracted (using 70% ethanol, final concentration) immediately upon thawing; iii) subjected to two freeze–thaw cycles and extracted immediately upon second thawing; iv) subjected to three freeze–thaw cycles and extracted immediately upon third thawing; v) kept on ice for 1 h and extracted immediately after; vi) kept on ice for 3 h and extracted immediately after; vii) kept without ice for 1 h at RT and extracted immediately after; viii) kept without ice for 3 h and extracted immediately after. The RT was measured with a digital thermometer every 30 min during the study and averaged 23.4 °C.

Later, an additional plasma pool was prepared of fresh plasma from some (*n*=5) of the healthy subjects contributing to the first pool, spiked with glucagon (0 and 40 pmol/l, final concentration), and aliquoted. Thereafter, the valiquots were kept on ice for up to 24 h. These aliquots (*N*=6–8 for each time point) were extracted after 1, 3, 6, 12, and 24 h, respectively, measured (see below) and compared with a set of aliquots extracted immediately after spiking with peptide. The same procedure was followed for a pool of spiked and aliquoted assay buffer (80 mmol/l sodium phosphate buffer, pH 7.5, containing in addition 0.1% wt/vol human serum albumin, 10 mmol/l EDTA, and 0.6 mmol/l thimerosal: Cat No. T-5125, Sigma Chemical Co.). After extraction, peptide concentrations were analyzed using the assays described below.

#### The effect of short-term storage conditions on the activity of dipeptidyl-peptidase 4

Plasma (new pool, *n*=5) prepared from blood sampled with or without the addition of the DPP4 inhibitor valine pyrrolidide (0.01 mM final concentration) was aliquoted (5 μl) into 96-well plates, and incubated on ice or at RT for 0, 1, 2, and 3 h. DPP4 activity was assessed kinetically by standard procedures (9) using Gly-Pro-*p*-nitroaniline (pNa; 1 mmol/l, Bachem, Cat. No. L1880) as substrate and *p*-nitroaniline (pNa) as standard (Sigma–Aldrich, Cat. No. N1218). pNa accumulation was monitored at 405 nm by spectrophotometry. Enzyme activity was calculated as mU/ml, where one unit of activity is defined as the amount of enzyme which cleaves 1 μmol substrate/min.

#### The effect of different enzyme inhibitors on the stability of GLP-1 and glucagon

Three different enzyme inhibitors (DPP4 inhibitor: 0.1 mmol/l valine pyrrolidide; NEP 24.11 inhibitor: 3.5 μmol/l phosphoramidon, Cat. No. 119942-99, Santa Cruz; or a proprietary cocktail mixture of protease and esterase inhibitors: 1 mg/ml Pefabloc Cat. No. 11873601001, Roche Diagnostics GmbH) were separately added to three aliquoted portions of a human plasma pool (new plasma pool, *n*=5) and to assay buffer. GLP-1 and glucagon (0 and 40 pmol/l) were added to the portions, whereafter they were stored at either room temperature or on ice for 1 h and measured (as described below plasma samples after extraction and buffer samples directly).

### The effect of long-term storage conditions on the stability of GLP-1 and glucagon

From each of the original spiked plasma portions, eight to ten aliquots were distributed into low-adsorption Nunc tubes (VWR-Bie&Berntsen A/S, Herlev, Denmark). One set of samples (*N*=8–10) was extracted and analyzed immediately to serve as controls. The remaining eight sets were divided into two and stored at −20 °C or −80 °C. Samples (*N*=8–10) from each set were analyzed after 1, 3, 6, and 12 months of storage. In addition, seven portions of the new plasma pool as well as assay buffer (to which was added 6% human serum albumin (HSA) to mimick plasma), spiked with 0 and 40 pmol/l of glucagon (*N*=5 for each set of spiked samples), were assayed (plasma after extraction) after freezing (at −20 °C) for 0 (i.e., they were measured immediately without freezing), 1, 3, 6, 12, 24, and 72 h of storage at −20 °C.

### Measurements

Glucagon was measured using in-house RIAs, specific for either the intact C-terminus (codename 4305) or the N-terminus (codename 4830) of the molecule, as previously described (10, 11). GLP-1 7–36NH_2_ was measured with both an in-house RIA specific for the amidated C-terminal (total GLP-1 assay, codename 89390 (12)) and with an in-house two-site sandwich ELISA for GLP-1, using a modification of the method originally described by Wilken *et al*. (13), as described in Supplementary 1, see section on supplementary data given at the end of this article. With this modification, the sensitivity of the assay is around 1 pmol/l and the intra-assay variation is below 5%, similar to that of the other assays. Assays have previously been evaluated according to the Richterich criteria as well CLSI (Clinical and Laboratory Standards Institute) guidelines for Immunoassay (I/LA23-A, I/LA21-A2, and EP24-A) (7, 8, 11).

Assay setup was automated with a robotic pipetting system MultiPROBE 204 Packard and Janus (PerkinElmer, Waltham, MA, USA) to minimize differences between measurements.

### Calculations and statistics

The recovery in percent was calculated as the measured concentration in the individual, spiked plasma sample minus the concentration in the corresponding non-spiked plasma sample divided by the theoretical spiked concentration of peptide and multiplied by 100. For the presentation of data, we normalized the recovery after each treatment by adjusting for the recovery of the samples extracted immediately, set as 100% (Table 1). Differences between treatments were analyzed by one-way ANOVA for repeated measurement with *post hoc* Bonferroni correction. For DPP4 activity, a paired Student's *t*-test was used. A *P*<0.05 was considered to be statistically significant. Statistical analysis was carried out using GraphPad Prism version 6.00 for Windows, GraphPad Software, La Jolla, CA, USA, www.graphpad.com.

## Results

### Recovery of GLP-1 and glucagon

The recovery of GLP-1 7–36NH_2_, 9–36NH_2_, and glucagon added to human plasma and subjected to immediate extraction and assay was evaluated by measuring samples spiked with two concentrations (10 and 40 pmol/l) of each peptide (*n*=21 for each peptide and concentration). As given in Table 1, recovery of all three peptides was around 70%. These results are similar to previously observed recoveries. In model experiments using assay with 6% HSA (mimicking the matrix effect in plasma; the unspecfic interference of plasma components on measurement of e.g. GLP-1 and glucagon), the recovery after extraction was also about 70% representing the inherent loss incurred by the extraction procedure (shown Supplementary Figure 1, see section on supplementary data given at the end of this article). The recovery of the peptides measured immediately after spiking of unfrozen plasma was therefore set to represent 100% recovery.

### Effect of short-term time handling and temperature on stability of GLP-1 and glucagon

**Figure 1:**
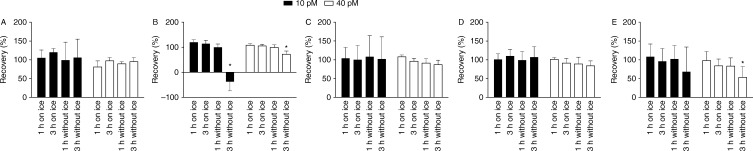
The impact of storage conditions with or without ice for 1 and 3 h, respectively, on stability of added (10 and 40 pmol/l) GLP-1 and glucagon in human plasma to which DPP4 inhibitor and aprotinin were added. (A) 7–36NH_2_ GLP-1 isoform spiked in human plasma and measured with a C-terminal-specific RIA. (B) 7–36NH_2_ GLP-1 isoform spiked in human plasma and measured with a sandwich ELISA specific for intact GLP-1. (C) 9–36NH_2_ GLP-1 isoform spiked in human plasma and measured with a C-terminal-specific RIA. (D) Glucagon spiked in human plasma and measured with a C-terminal RIA. (E) Glucagon spiked in human plasma and measured with an N-terminal RIA. Each result represents mean±s.d. of six replicated determinations of GLP-1 and glucagon measured with different assays and normalized to results measured after immediate extraction. *P<0.05 tested by one-way ANOVA for repeated measurement with *post hoc* Bonferroni correction. Standard curve CV ranging from 4 to 10%.

The concentrations of GLP-1 (7–36NH_2_) measured using the C-terminal RIA were relatively stable over short-term periods up to 3 h, irrespective of whether samples were standing at RT or placed on ice, with full recovery for samples spiked with both 10 and 40 pmol/l (Fig. 1A). Similarly, intact GLP-1 levels (determined by sandwich ELISA) were also stable (in plasma to which a DPP4 inhibitor had been added) for up to 3 h on ice or for 1 h at RT (Fig. 1B). However, when samples were left at RT for 3 h, recovery of the intact peptide was significantly (*P*=0.0001) reduced (Fig. 1B).

The GLP-1 metabolite, GLP-1 (9–36NH_2_), was also relatively stable over the short-term, with concentrations measured with the C-terminally directed RIA not changing significantly, even in the samples left at RT for 3 h (Fig. 1C). However, it was evident that at the lower concentration (10 pmol/l spiked samples), the variability of the recovery for samples standing at RT increased significantly (*P*=0.03) from ±33% to ±72% (Fig. 1C).

Glucagon concentrations were not significantly influenced by storage on ice for up to 24 h, independent of assay (Fig. 1D, E and Supplementary Figure 2C, D, see section on supplementary data given at the end of this article). Glucagon also seemed to be relatively stable at RT for 1 h, although by 3 h a significant (*P*=0.02) reduction in recovery was observed with the N-terminal-specific assay (Fig. 1E).

#### Effect of temperature on plasma DPP4 activity

**Figure 2:**
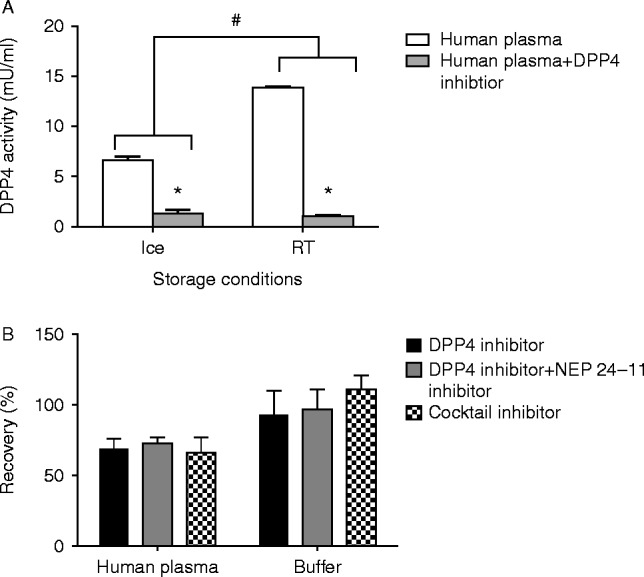
(A) DPP4 activity in human plasma on ice (left) or at RT (right) with (grey bars) or without (white bars) addition of DPP4 inhibitor (valine pyrrolidide 0.01 mM). *P<0.05 between treatment and ^#^ *P*<0.05 between samples on ice and RT tested with a Student's paired *t*-test. Each result represents mean±s.d. of eight replicated determinations. (B) Enzyme inhibitors illustrates the recovery (%) of intact GLP-1 in human plasma (left part) or in buffer (right part) treated with respectively DPP4 inhibitor (black), DPP4 inhibitor+NEP 24.11 inhibitor (grey), and an inhibitor cocktail (grey with dots). No significant changes in recovery between treatments but as expected between human plasma and buffer (matrix effect and solvent extraction of human plasma). Samples were kept at RT for 1 h. Similar data were obtained when samples were kept on ice (data not shown). Each result represents mean±s.d. of eight replicated determinations. Standard curve CV ranging from 5 to 9%.

DPP4 activity in plasma placed on ice was significantly reduced compared with the activity when samples were placed at RT (Fig. 2A). Addition of valine pyrrolidide further inhibited DPP4 activity under both storage conditions up to ∼90% (both *P*<0.05).

#### Effect of enzyme inhibitors on GLP-1 and glucagon

The combination of a NEP 24.11 inhibitor and a DPP4 inhibitor or the use of an inhibitor cocktail Pefabloc did not significantly (*P*=0.43) increase the recovery of GLP-1 compared with the use of a DPP4 inhibitor alone when samples were kept at RT for 1 h (Fig. 2B). Similar data were obtained for glucagon or when samples were placed on ice (data not shown).

### Stability of GLP-1 and glucagon upon freezing–thawing cycles

**Figure 3:**
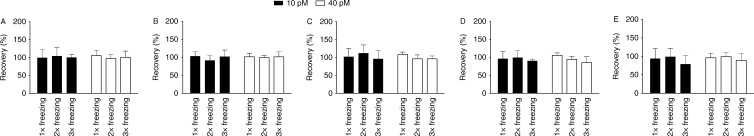
The impact of refreezing cycles on recovery of added (10 and 40 pmol/l) GLP-1 and glucagon in human plasma. (A) 7–36NH_2_ GLP-1 isoform spiked in human plasma and measured with a C-terminal-specific RIA. (B) 7–36NH_2_ GLP-1 isoform spiked in human plasma and measured with a sandwich ELISA specific for intact GLP-1. (C) 9–36NH_2_ GLP-1 isoform spiked in human plasma and measured with a with a C-terminal-specific RIA. (D) Glucagon spiked in human plasma and measured with a C-terminal-specific RIA. (E) Glucagon spiked in human plasma and measured with a N-terminal-specific RIA. Each result represents mean±s.d. of six replicated determinations of GLP-1 and glucagon measured with different assays and normalized to samples extracted immediately after peptide addition. Standard curve CV ranging from 4 to 10%.

Concentrations of GLP-1 7–36NH_2_ and 9–36NH_2_ were not significantly changed by up to three repeated freeze–thaw cycles, independent of assay used (Fig. 3A, B, and C).

For glucagon, although concentrations did not differ significantly, there may be a trend (C-terminal assay 10 pmol/l: *P*=0.12, C-terminal assay 40 pmol/l: *P*=0.19, N-terminal assay 10 pmol/l: *P*=0.16, C-terminal assay 40 pmol/l: *P*=0.21) toward lower mean concentrations after the third cycle of refreezing, compared with samples extracted immediately, using C- and N-terminal specific RIAs (87 and 85% respectively; Fig. 3D and E).

### Long-term storage conditions

**Figure 4:**
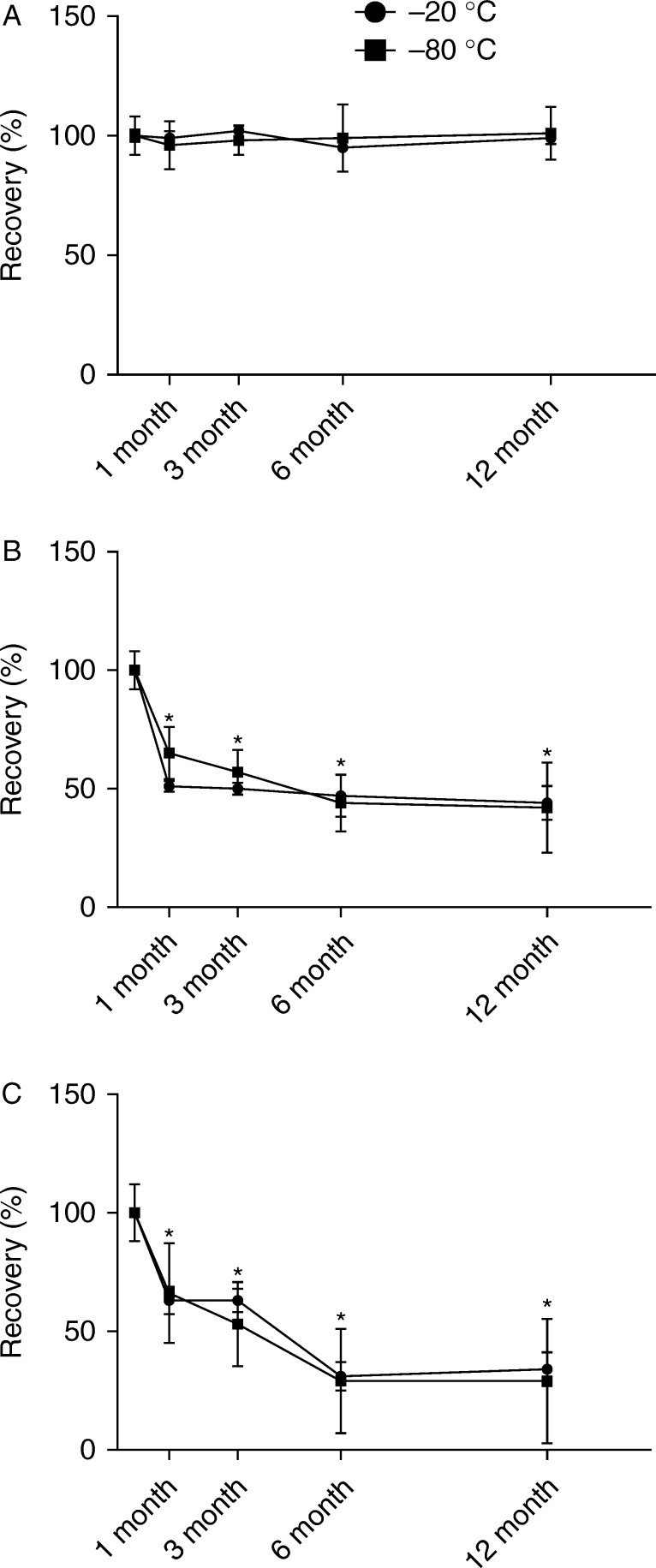
The impact of long-term storage at −20 °C (black circle) and at −80 °C (black square), respectively, on the recovery of added (40 pmol/l) GLP-1 and glucagon in human plasma. (A) 7–36NH_2_ GLP-1 isoform spiked in human plasma and measured with a sandwich ELISA specific for intact GLP-1. (B) Glucagon spiked in human plasma and measured with a C-terminal-specific RIA. (C) Glucagon spiked in human plasma and measured with a N-terminal-specific RIA. Each result represents mean±s.d. of eight replicated determinations of GLP-1 and glucagon measured with different assays and normalized to result from samples extracted immediately after addition. *P<0.05 tested by one-way ANOVA for repeated measurement with *post hoc* Bonferroni correction. 10 pmol/l data not shown but was similar. Standard curve CV ranging from 6 to 13%.

Intact GLP-1 appeared stable over long-term storage, with concentrations remaining unchanged for up to 12 months, independent of storage temperature (−80 compared with −20 °C; Fig. 4A). Similar results were seen for 9–36NH_2_ (data not shown). In contrast, glucagon concentrations were significantly (*P*=0.0003) reduced by long-term storage at both −20 and −80 °C. Recovery, measured with both C- and N-terminal assays dropped by almost half after 1 month, continuing to fall more slowly over the subsequent 5 months before stabilizing for the remainder of the 12 months of the study (Fig. 4B and C). The results were surprising and therefore we repeated the experiment using a new plasma pool to investigate at which time the loss in recovery of glucagon occurs. As shown in Supplementary Figure 2A and B, the recovery of glucagon in human plasma was significantly (*P*=0.0004) reduced, compared with results of immediate extraction and measurement (but no freezing at all), after 3 h of freezing, whereas the recovery in buffer seemed unaffected for up to 72 h. Similar findings were obtained using C- and N-terminal assays.

## Discussion and conclusion

GLP-1 and glucagon are important regulators of glucose homeostasis and are involved in the pathogenesis of T2DM (14). Therefore, assessments of their plasma concentrations are often part of clinical studies (11, 15, 16). Given the long-term nature of many of these studies, plasma samples are, by necessity, often kept in freezers for prolonged periods (6 months or more) before analysis. Moreover, the way in which collected plasma samples are treated before long-term storage may also vary between studies. The effects of sample handling or storage condition on the stability of these peptides have never been thoroughly investigated.

The results of this study show that GLP-1 in human plasma samples supplemented with a DPP4 inhibitor appears to be relatively stable over the short-term. Thus, concentrations determined with the C-terminal RIA did not change significantly over the 3-h period, whether the samples were kept on ice or left at RT. Such assays are useful for determining ‘total’ GLP-1 levels (12), because they detect not only the intact peptide but also the N-terminally truncated metabolite which arises from DPP4 cleavage, as well as any smaller peptide fragments, provided that the C-terminal epitope remains intact. Intact GLP-1 levels (the full sequence as measured with the sandwich ELISA) also remained unchanged for up to 3 h in DPP4 inhibitor-treated plasma samples stored on ice, and even at RT, concentrations did not drop significantly for the first hour. However, by 3 h, levels measured with the ELISA decreased significantly. One explanation could be that the DPP4 inhibitor employed in this study (valine pyrrolidide) was unable to fully prevent N-terminal degradation occurring when plasma was left for more than 1 h at RT. We therefore investigated plasma DPP4 activity and found that simply chilling samples in ice was sufficient to reduce DPP4 activity by 52% compared with RT, while the addition of the DPP4 inhibitor reduced levels further (to 90%), although not completely. Given that intact GLP-1 was apparently stable in the samples on ice (levels determined by ELISA did not change), this would suggest that at these low levels of DPP4 activity, any cleavage occurs at a slow rate and is not detectable over the 3-h time frame. Alternatively, it should be recognized that the chromogenic substrate used to assess DPP4 activity (pNa) is not specific for only DPP4. It is, therefore, possible that other N-terminally cleaving proteases, not inhibitable by the DPP4 inhibitor used here (valine pyrrolidide) may still be active. Whether such enzymes are able to cleave physiological substrates such as GLP-1 is unknown. Taken together, these findings suggest that although GLP-1 levels may be stable if plasma is chilled promptly, it is prudent to collect blood samples into tubes containing a DPP4 inhibitor when intact GLP-1 levels are to be determined. This could be particularly important in clinical trials, where immediate centrifugation and freezing of plasma samples may not always be possible.

Other enzymes such as endopeptidase 24.11 (NEP 24.11) have previously been suggested to be responsible for GLP-1 degradation in pigs *in vivo* (17), and the use of inhibitor cocktails has been promoted for both rodent and human studies (18, 19). However, it is unknown whether the activity of other enzymes in plasma is sufficient to affect peptide levels after sampling. We therefore studied whether the inclusion of a NEP 24.11 inhibitor or a commonly used inhibitor cocktail (Pefabloc) would be beneficial with regards to preservation of intact GLP-1 in human plasma. This was demonstrated not to be the case with neither inhibitor improving the recovery of intact GLP-1 beyond that obtained with a DPP4 inhibitor alone, suggesting that GLP-1 degradation in samples *ex vivo* is mainly due to DPP4, at least in the short-term. This interpretation is further supported by the observation that comparable recoveries are obtained when blood is collected in the presence of a DPP4 inhibitor alone or into commercially available tubes which are pre-coated with a variety of protease inhibitors (metabolic biomarker BD tubes P700 and P800; data not shown).

The recoveries of glucagon measured with a C-terminal specific assay were relatively stable up to 3 h, both when human plasma was stored on ice as well at RT. Furthermore, glucagon seems stable if samples are stored on ice for up to 24 h (shown in Supplementary Figure 2C and D). In contrast, when glucagon levels were analyzed using an N-terminal specific assay, recoveries dropped significantly after 3 h incubation at RT. This is unlikely to be explained by the activity of DPP4 because a DPP4 inhibitor was added in agreement with previous studies indicating that glucagon is a poor *in vivo* substrate for DPP4 (4).

It is usually recommended to avoid thawing and re-freezing plasma samples before analysis, because this has been assumed to have a detrimental effect on the stability of peptide hormones. Somewhat to our surprise, we found that stability of GLP-1, the metabolite, and the intact peptide, was not significantly affected, with no loss of recovery after three freeze–thaw cycles. Moreover, when handled correctly (samples kept chilled before centrifugation and freezing and – for intact GLP-1 – collected in the presence of a DPP4), GLP-1 was also stable over longer term storage of up to 1 year. Unexpectedly, glucagon showed a greater reduction in recovery degradation than GLP-1 with an almost 50% loss after 1 month (shown in Fig. 1E, Fig. 4A and B, and Supplementary Figure 2A and B). This reduction seems unrelated to the mere act of freezing and thawing (where plasma was frozen for up to 1 h), but was nevertheless quite dramatic after only 3 h of freezing again with a loss of about 50% (shown in Supplementary Figure 2A and B) and recovery continued to drop until 6 months regardless of storage temperature (shown in Fig. 4A and B). The significant reduction in recovery of glucagon was only observed in plasma and not in buffer (spiked with 6% human albumin mimicking the plasma matrix), suggesting that it is unlikely to be caused by non-specific adsorption by the tube material but indicates that it is a plasma-related event. Whether the decrease is related to the similar decrease observed after storage without freezing at room temperature for >3 h cannot be determined from these data. It is known that glucagon may show conformational changes (e.g. fibrillation) that might reduce the concentrations of free peptide in solution, and this might provide an explanation. In clinical studies, plasma samples are rarely measured immediately after blood sampling and plasma preparation, therefore our data indicate the measurements in general are underestimated. Further studies investigating whether loss is constant for all samples (in pmol/l) or varies with concentration are therefore necessary.

In conclusion, combination of DPP4 inhibition and storage of samples on ice provide adequate protection of intact and total GLP-1 with regards to both short-term storage (including freeze–thaw cycles) and long-term storage (12 months). In contrast, glucagon seems susceptible to long-term storage, which theoretically may have implications for interpretation (underestimation) of glucagon levels in clinical studies. Future studies may address whether the apparent instability of glucagon in human plasma upon freezing is caused by enzymatic degradation or nonenzymatic process (e.g., fibrillation).

## Supplementary data

This is linked to the online version of the paper at http://dx.doi.org/10.1530/EC-14-0126.

## Author contribution statement

M J Bak, J J Holst, C F Deacon, R E Kuhre, L W Christensen, B Hartmann, and N J Wewer Albrechtsen planned and designed the study; M J Bak, R E Kuhre, and N J Wewer Albrechtsen ran the experiments; N J Wewer Albrechtsen and R E Kuhre ran the analyses; N J Wewer Albrechtsen, M J Bak, B Hartmann, C F Deacon, and J J Holst interpreted the results; N J Wewer Albrechtsen and M J Bak wrote the manuscript; B Hartmann, C F Deacon, J J Holst, R E Kuhre, L W Christensen revised the manuscript; all authors approved the final version of the manuscript.

## Figures and Tables

**Table 1:** Each result represents mean±s.d. of measured glucagon-like peptide 1 (7–36NH_2_ and 9–36NH_2_) and glucagon in a human plasma (*n*=21 for each concentration).

	**Theoretical concentration** (pmol/l)	**Mean±s.d.**				
		GLP-1 7–36NH_2_		GLP-1 9–36NH_2_	Glucagon	
C-term	Intact	C-term	C-term	N-term
Immediately extraction/basal conditions (pmol/l)	10	6.9±0.4	7.2±0.3	7.1±0.9	7.7±0.8	6.5±1
40	30±3	32±1.9	31±3.8	28.6±1.6	26.7±3.5
Immediately extraction/basal conditions (%)	10	69±5	72±4	71±12	77±10	65±15
	40	75±10	80±6	77±12	72±6	67±13
